# Antioxidant Peptides from Monkfish Swim Bladders: Ameliorating NAFLD In Vitro by Suppressing Lipid Accumulation and Oxidative Stress via Regulating AMPK/Nrf2 Pathway

**DOI:** 10.3390/md21060360

**Published:** 2023-06-16

**Authors:** Ming-Feng Wu, Qing-Hao Xi, Yan Sheng, Yu-Mei Wang, Wan-Yi Wang, Chang-Feng Chi, Bin Wang

**Affiliations:** 1 Zhejiang Provincial Engineering Technology Research Center of Marine Biomedical Products, School of Food and Pharmacy, Zhejiang Ocean University, Zhoushan 316022, China; wu15873772658@163.com (M.-F.W.); xiqinghao2022@163.com (Q.-H.X.); 18223101235@163.com (Y.S.); wangyumei731@163.com (Y.-M.W.); 2 National and Provincial Joint Laboratory of Exploration and Utilization of Marine Aquatic Genetic Resources, National Engineering Research Center of Marine Facilities Aquaculture, School of Marine Science and Technology, Zhejiang Ocean University, Zhoushan 316022, China; xxx0001112223@163.com

**Keywords:** monkfish (*Lophius litulon*), swim bladders, antioxidant peptide, nonalcoholic fatty liver disease (NAFLD), AMPK/Nrf2 pathway

## Abstract

In this study, we investigate the ameliorating functions of QDYD (MSP2), ARW (MSP8), DDGGK (MSP10), YPAGP (MSP13) and DPAGP (MSP18) from monkfish swim bladders on an FFA-induced NAFLD model of HepG2 cells. The lipid-lowering mechanisms revealed that these five oligopeptides can up-regulate the expression of phospho-AMP-activated protein kinase (*p*-AMPK) proteins to inhibit the expression of the sterol regulatory element binding protein-1c (SREBP-1c) proteins on increasing lipid synthesis and up-regulating the expression of the PPAP-α and CPT-1 proteins on promoting the β-oxidation of fatty acids. Moreover, QDYD (MSP2), ARW (MSP8), DDGGK (MSP10), YPAGP (MSP13) and DPAGP (MSP18) can significantly inhibit reactive oxygen species’ (ROS) production, promote the activities of intracellular antioxidases (superoxide dismutase, SOD; glutathione peroxidase, GSH-PX; and catalase, CAT) and bring down the content of malondialdehyde (MDA) derived from lipid peroxidation. Further investigations revealed that the regulation of these five oligopeptides on oxidative stress was achieved through activating the nuclear factor erythroid 2-related factor 2 (Nrf2) pathway to raise the expression levels of the heme oxygenase 1 (HO-1) protein and downstream antioxidant proteases. Therefore, QDYD (MSP2), ARW (MSP8), DDGGK (MSP10), YPAGP (MSP13) and DPAGP (MSP18) could serve as candidate ingredients to develop functional products for treating NAFLD.

## 1. Introduction

Non-alcoholic fatty liver disease (NAFLD) is characterized by steatosis, liver inflammation, hepatocellular damage and progressive fibrosis and is the main reason for increasing chronic liver disease in world [1,2,3]. As the commonest chronic hepatism, NAFLD seriously affects people’s health and is closely relevant to metabolic disturbance, such as hyperglycemia, central obesity, insulin resistance, lipid metabolism disorder, adult-onset diabetes, hypertension and persistent abnormal liver functions [3,4,5]. Therefore, the global prevalence of NAFLD is presumed to be approximately 25%, increasing from 13% in Africa to 42% in Southeast Asia [5,6,7]. The prevalence of NAFLD is predicted to increase by up to 56% in China, the United Kingdom, Germany, the United States and Japan by 2030 [1].

There is a kinetic equilibrium relation between lipid synthesis and degradation in the liver, and it will result in a large accumulation of lipids if this balance relation is disrupted, giving rise to oxidative stress and hepatic steatosis [8,9]. The imbalance between antioxidant contents and reactive oxygen species’ (ROS) production in vivo and the high lipid content in livers gives rise to a variety of metabolic dysfunctions [8,10]. Therefore, inhibiting lipid synthesis and/or increasing lipid degradation are taken as effective methods for lowering lipid accumulation in livers. In addition, inhibition or elimination of ROS-induced oxidative injury also plays a key role in reducing NAFLD incidence [11,12,13]. Therefore, it is vital to reduce oxidative stress levels and balance the lipid metabolism to reduce NAFLD incidence.

In addition to adjusting personal lifestyles, such as losing weight, physical exercise and a Mediterranean diet, some synthetic drugs including Fenofibrate and Pioglitazone are considered to treat NAFLD, but these drugs have caused some side effects [8,10,14]. Therefore, there is a strong need to find safer and more effective alternative drugs for NAFLD. Compared to synthetic drugs, some natural ingredients, such as curcumin, carotenoid, quercetin, resveratrol, gastrodigenin, berberine and peptides, demonstrate remarkable antioxidant, anti-diabetic and anti-hyperlipidemia properties and are considered to serve as dietary additives to treat NAFLD [14,15,16,17]. 

Bioactive peptides (BPs) comprise 3–30 amino acid residues with molecular weights (MWs) ranged from 500 to 1850 Da and are generated from diversified protein resources by enzymatic hydrolysis, chemical degradation and microbial fermentation methods [18,19,20,21]. In addition to their widely accepted nutritional value, BPs have also been proven to have important applications in promoting human health due to their significant physiological and pharmacological functions [22,23,24,25]. What is more noteworthy is that some BPs have remarkable lipid-lowering and antioxidant activities and show significant superiority in curing NAFLD [26,27,28]. For example, the murine peptide hormone of metabolitin (MTL) can greatly improve the clinical symptoms of NAFLD via controlling lipid metabolism and insulin resistance [27]; VIAPW and IRWWW from the muscle hydrolysate of *Miichthys miiuy* can decrease the levels of intracellular triglyceride (TG) content © and total cholesterol (TC) [11]; tetra peptide (VHVV) from soy has exhibited a high preventive effect on the liver dysfunction caused by hyperglycemia [29]; and hydrolysates of sea cucumber gonads [30], *Octopus vulgaris* [31] and *Mucuna pruriens* [32] have presented anti-hyperlipidemic effects. Peptide fraction (MW < 1 kDa) from monkfish muscles can increase the antioxidant ability in livers to prevent NAFLD progression mainly by modulating the intestinal flora and AMPK/Nrf2 pathways [33,34]. Therefore, BPs have attractive potential in functionality foods, nutraceutical supplements and pharmaceutical products for treating NAFLD [35,36,37].

Monkfish (*Lophius litulon*) belongs to the members of the genus *Lophiidae* and is found mainly in the Northwest Pacific Ocean [38]. Presently, BPs from monkfish muscles and its processing by-products showed significant bioactivities, such as radical scavenging activities [38,39], cytoprotection for H_2_O_2_-damaged HepG2 cells [40], enhancing the immune regulatory effect [41], ameliorating high-fat-diet-induced nephrotoxicity [34], anti-fatigue effects [42], antihypertensive abilities [43] and protection against chronic kidney injuries [44]. In addition, eighteen antioxidant peptides (MSP1–MSP18) were purified from the swim bladder hydrolysate of monkfish and identified as Tyr-Asp-Tyr-Asp (YDYD, MSP1); Gln-Asp-Tyr-Asp (QDYD, MSP2); Ala-Gly-Pro-Ala-Ser (AGPAS, MSP3); Gly-Pro-Gly-Pro-His-Gly-Pro-Ser-Gly-Pro (GPGPHGPSGP, MSP4); Gly-Pro-Lys (GPK, MSP5); His-Arg-Glu (HRE, MSP6); Gly-Arg-Trp (GRW, MSP7); Ala-Arg-Trp (ARW, MSP8); Gly-Pro-Thr-Glu (GPTE, MSP9); Asp-Asp-Gly-Gly-Lys (DDGGK, MSP10); Ile-Gly-Pro-Ala-Ser (IGPAS, MSP11); Ala-Lys-Pro-Ala-Thr (AKPAT, MSP12); Tyr-Pro-Ala-Gly-Pro (YPAGP, MSP13); Asp-Pro-Thr (DPT, MSP14),; Phe-Pro-Gly-Pro-Thr (FPGPT, MSP15); Gly-Pro-Gly-Pro-Thr (GPGPT, MSP16); Gly-Pro-Thr (GPT, MSP17); and Asp-Pro-Ala-Gly-Pro (DPAGP, MSP18), respectively [45]. As a consequence, the objective of this investigation was to research and illuminate the ameliorating functions and mechanisms of these isolated antioxidant peptides (MSP1–MSP18) from monkfish swim bladders on NAFLD using free fatty acid (FFA)-induced HepG2 cells. Thus, it provides a theoretical basis for monkfish swim bladder antioxidant peptides as antioxidants in NAFLD health promotion products.

## 2. Results

### 2.1. Establishment of FFA-Induced NAFLD Model of HepG2 Cells

**Figure 1:**
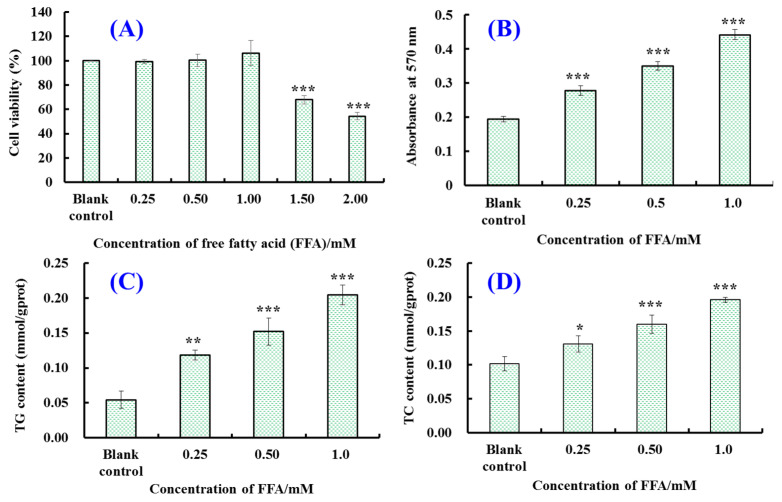
Influences of free fatty acids (FFA) on cell viability (**A**), intracellular lipid accumulation (**B**), triglyceride (TG) content (**C**) and total cholesterol (TC) content (**D**) in HepG2 cells. All data are presented as the mean ± SD of triplicate results. *** *p* < 0.001, ** *p* < 0.01 and * *p* < 0.05 vs. blank control group.

Figure 1A indicates that the viability of HepG2 cells showed a slow upward trend with FFA concentration, ranging from 0 to 1.0 mM. However, the viability of HepG2 cells treated with 1.5 mM FFA decreased to 67.82 ± 3.30%, markedly different from that of the blank group (*p* < 0.001). This demonstrates that excess FFA lead to the apoptosis of HepG2 cells. Therefore, the FFA concentration ranged from 0.25 to 1.0 mM was chosen for establishing the NAFLD model of HepG2 cells.

**Figure 2:**
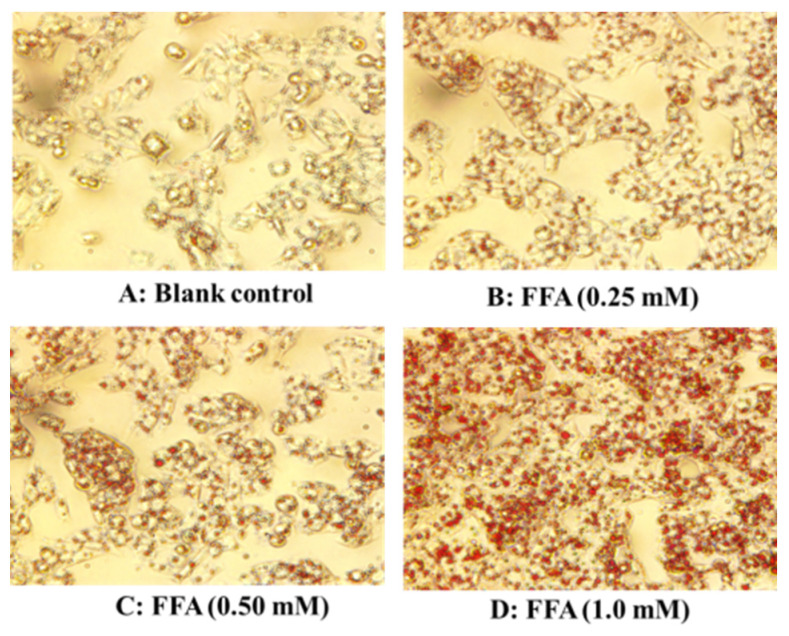
Effects of FFA (0–1.0 mM) on morphological characteristics of HepG2 cells (×20): (**A**) blank control; (**B**) FFA (0.25 mM); (**C**) FFA (0.50 mM); (**D**): FFA (1.0 mM).

The effects of FFA (0–1.0 mM) on lipid accumulation in HepG2 cells were analyzed by employing the Oil red O staining method (Figure 2). 

These morphological characteristics of HepG2 cells indicate that a small number of red lipid droplets were present in the blank control group, while the number of intracellular red lipid droplets increased significantly when the FFA concentration increased from 0.25 to 1.0 mM. After quantifying the images of Figure 2, it was found that the intracellular lipid contents in the 0.25, 0.50 and 1.0 mM FFA groups were markedly (*p* < 0.001) higher than the blank control group and were 1.45-, 1.80- and 2.28-fold of that in the blank group (Figure 1B).

Figure 1C,D show that the TG and TC contents in the HepG2 cells were raised gradually with the FFA concentration. When the FFA concentration was 1.0 mM, the TG content in the HepG2 cells increased to 0.20 ± 0.014 mmol/gprot, which was about 4-fold that of the blank group (0.054 ± 0.012 mmol/gprot), and the TC content increased to 0.20 ± 0.0037 mmol/gprot, which was about 2-fold of that in the blank group (0.10 ± 0.01 mmol/gprot). The data indicate that 0.25–1.0 mM FFA can significantly increase the TG and TC contents in HepG2 cells, resulting in lipid accumulation. Therefore, 1.0 mM FFA was selected to establish the NAFLD model of the HepG2 cells.

### 2.2. Screening the Peptides with High Hypolipidemic Activity from MSP1–MSP18

**Figure 3:**
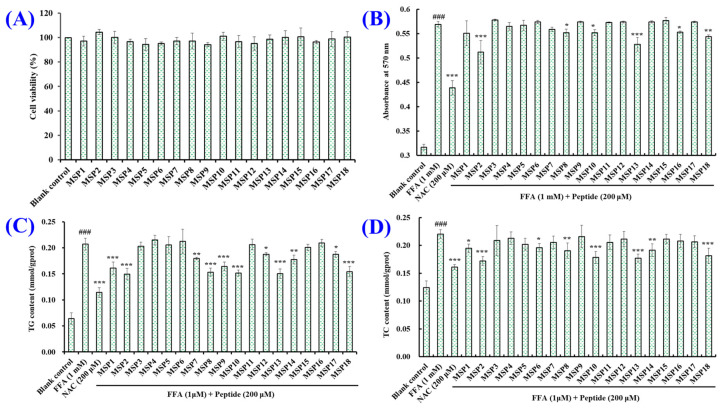
Effects of eighteen antioxidant peptides (MSP1–MSP18) on the viability of HepG2 cells (**A**) and intracellular lipid accumulation (**B**), and the TG (**C**) and TC (**D**) contents in FFA-induced NAFLD model of HepG2 cells after 24 h of treatment. NAC at 200 µM was served as the positive control. All data are presented as the mean ± SD of triplicate results. ^###^ *p* < 0.001 vs. blank control group; *** *p* < 0.001, ** *p* < 0.01 and * *p* < 0.05 vs. FFA-induced NAFLD model of HepG2 cells.

In a previous report, antioxidant peptides from the protein hydrolysate of monkfish swim bladders presented a significantly cytoprotective function to H_2_O_2_-induced HepG2 cells by increasing cell viability at 200 μmol/L [45]. Therefore, a peptide concentration of 200 μM was used for researching the effects of MSP1–MSP18 on the viability of HepG2 cells (Figure 3A). At 200 μM, the viability of HepG2 cells treated with MSP1, MSP5, MSP6, MSP9 and MSP12 was lower than those of the blank control. The viability of HepG2 cells treated with MSP2 was 104.47 ± 2.03%, which was the maximum cell viability among the blank control and eighteen peptides group. What is more, there were no significant differences between the blank control group and peptides groups (*p* > 0.05). Therefore, the eighteen isolated Aps (MSP1–MSP18) display the possibility for developing liver-protective products due to their minimal effects on the proliferation of HepG2 cells.

**Figure 4:**
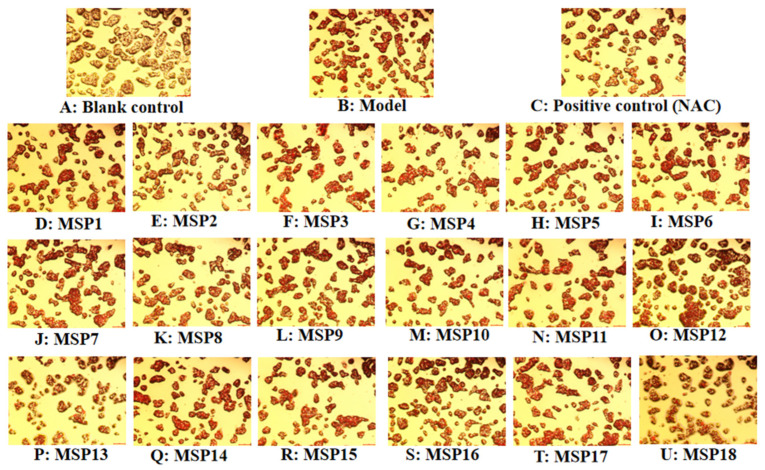
Effects of antioxidant peptides (MSP1–MSP18) on morphological characteristics of FFA-induced non-alcoholic fatty liver disease (NAFLD) model of HepG2 cells (20×). N-Acetyl-L-cysteine (NAC) was used as a positive control.

Figure 3B and Figure 4 show that the amount and absorbance of red lipid droplets in the model group were significantly higher than that in the blank group (*p* < 0.001), demonstrating a successful establishment of the NAFLD cell model. Treating with MSP1–MSP18, the amount of red lipid droplets in the MSP1, MSP2, MSP7, MSP8, MSP10, MSP13, MSP14, MSP16 and MSP18 groups decreased significantly compared with the model group (*p* < 0.001, *p* < 0.01 or *p* < 0.05), but the contents of the red lipid droplets in the remaining nine peptide groups displayed no significant difference compared with the model group (*p* > 0.05). 

Figure 3C,D show that the influences of MSP1–MSP18 on the TG and TC contents in the FFA-induced NAFLD model of HepG2 cells and the TG and TC contents in 1.0 mM FFA-treated HepG2 cells were significantly increased by 2.23 and 0.77 times compared with the blank control group (*p* < 0.001). After incubation with MSP1–MSP18, the TG contents in the FFA-induced HepG2 cells showed a decreasing trend, except for MSP4, MSP6 and MSP16 (Figure 3C). In addition, MSP1, MSP2, MSP7, MSP8, MSP9, MSP10, MSP12, MSP13, MSP14, MSP17 and MSP 18 can significantly lower the TG content of FFA-induced HepG2 (*p* < 0.001). Figure 3D indicates that MSP1–MSP18 can reduce the contents of TC in FFA-induced HepG2 cells, and the TC contents in the MSP1, MSP2, MSP6, MSP8, MSP10, MSP13, MSP14 and MSP 18 groups were significantly lower than that in the model group (*p* < 0.001, *p* < 0.01 or *p* < 0.05). Therefore, MSP2, MSP8, MSP10, MSP13 and MSP 18 were selected to investigate the amelioration function and mechanism on NAFLD using the FFA-induced HepG2 model.

### 2.3. Hypolipidemic Activity of MSP2, MSP8, MSP10, MSP13 and MSP18 in FFA-Induced NAFLD Model of HepG2 Cells

**Figure 5:**
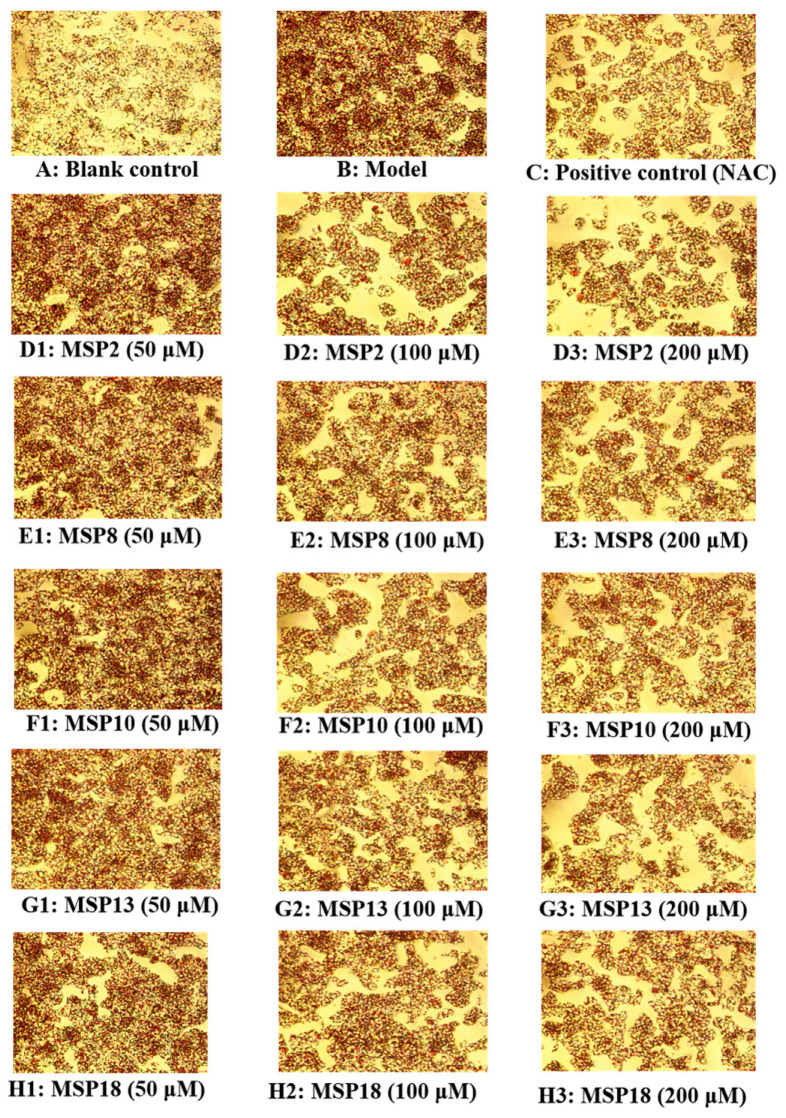
Effects of MSP2, MSP8, MSP10, MSP13 and MSP18 at 50, 100 and 200 µM on intracellular lipid accumulation in FFA-induced NAFLD model of HepG2 cells for 24 h (20×): (**A**) blank control; (**B**) FFA-induced cell model; (**C**) positive control (NAC); (**D1**–**D3**) MSP2 with the concentrations of 50, 100 and 200 μM, respectively; (**E1**–**E3**) MSP8 with the concentrations of 50, 100 and 200 μM, respectively; (**F1**–**F3**) MSP10 with the concentrations of 50, 100 and 200 μM, respectively; (**G1**–**G3**) MSP13 with the concentrations of 50, 100 and 200 μM, respectively; (**H1**–**H3**) MSP18 with the concentrations of 50, 100 and 200 μM, respectively.

**Figure 6:**
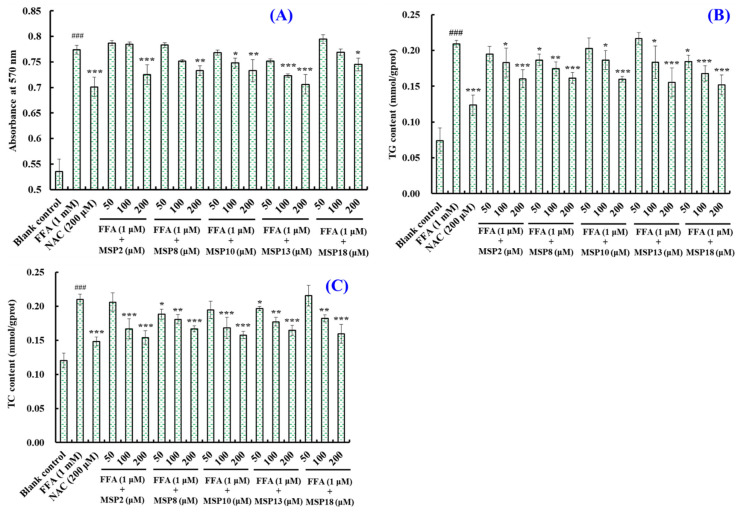
Effects of MSP2, MSP8, MSP10, MSP13 and MSP18 at 50, 100 and 200 µM on intracellular lipid accumulation (**A**), and the TG (**B**) and TC (**C**) contents in FFA-induced NAFLD model of HepG2 cells for 24 h. NAC at 200 µM was served as the positive control. All data are presented as the mean ± SD of triplicate results. ^###^ *p* < 0.001 vs. blank control group; *** *p* < 0.001, ** *p* < 0.01 and * *p* < 0.05 vs. FFA-induced NAFLD model of HepG2 cells.

The effects of low (50 µM), medium (100 µM) and high (200 µM) concentrations of MSP2, MSP8, MSP10, MSP13 and MSP18 on the lipid accumulation in the FFA-caused HepG2 cells were photographed and calculated by employing the Oil red O staining method (Figure 5 and Figure 6A). The absorbance of red lipid droplets in the model group was 1.45-fold of that in the blank group, demonstrating a successful establishment of the NAFLD cell model. In contrast to the model group, the intracellular lipid droplet amount in the peptide-treated groups was brought down with the rising of the peptide concentrations, indicating that the general tendency of lipid accumulation in HepG2 cells was gradually declining as the concentration increased in the MSP2, MSP8, MSP10, MSP13 and MSP18 groups. Moreover, MSP2 (100 μM) and MSP13 (100 and 200 μM) showed the strongest ability to reduce lipid accumulation in the FFA-induced NAFLD model of HepG2 cells (*p* < 0.001), followed by MSP8 (200 μM) and MSP10 (200 μM) (*p* < 0.01). In addition, MSP13 at 200 μM showed the same lipid-lowering ability as NAC.

The effects of low (50 µM), medium (100 µM) and high (200 µM) concentrations of MSP2, MSP8, MSP10, MSP13 and MSP18 on the TG and TC contents in FFA-induced HepG2 cells were measured and are displayed in Figure 6B,C. The TG and TC contents were 0.209 ± 0.005 and 0.210 ± 0.008 mmol/gprot in the FFA-induced HepG2 cells, which were 2.83- and 1.75-fold of those in the blank group. Within the measured concentrations, the intracellular TG and TC contents in the MSP2, MSP8, MSP10, MSP13 and MSP18 groups gradually decreased. Compared to the model group, MSP2, MSP8, MSP10, MSP13 and MSP18 at high (200 µM) concentrations and MSP18 at medium (100 µM) concentrations showed the strongest ability to reduce the TG content of the FFA-induced HepG2 cells (*p* < 0.001), followed by MSP8 at medium (100 µM) concentrations (*p* < 0.01). In addition, MSP2 (100 and 200 µM), MSP8 (200 µM), MSP10 (100 and 200 µM), MSP13 (200 µM) and MSP18 (200 µM) showed the strongest ability to reduce the TC content of the FFA-induced HepG2 cells (*p* < 0.001), followed by MSP8 (100 μM), MSP13 (100 µM) and MSP18 (100 µM) (*p* < 0.01). However, the lowering ability on the TC and TG quantity of MSP2, MSP8, MSP10, MSP13 and MSP18 was lower than that of NAC.

### 2.4. Antioxidant Activity of MSP2, MSP8, MSP10, MSP13 and MSP18 in FFA-Induced NAFLD Model of HepG2 Cells

**Figure 7:**
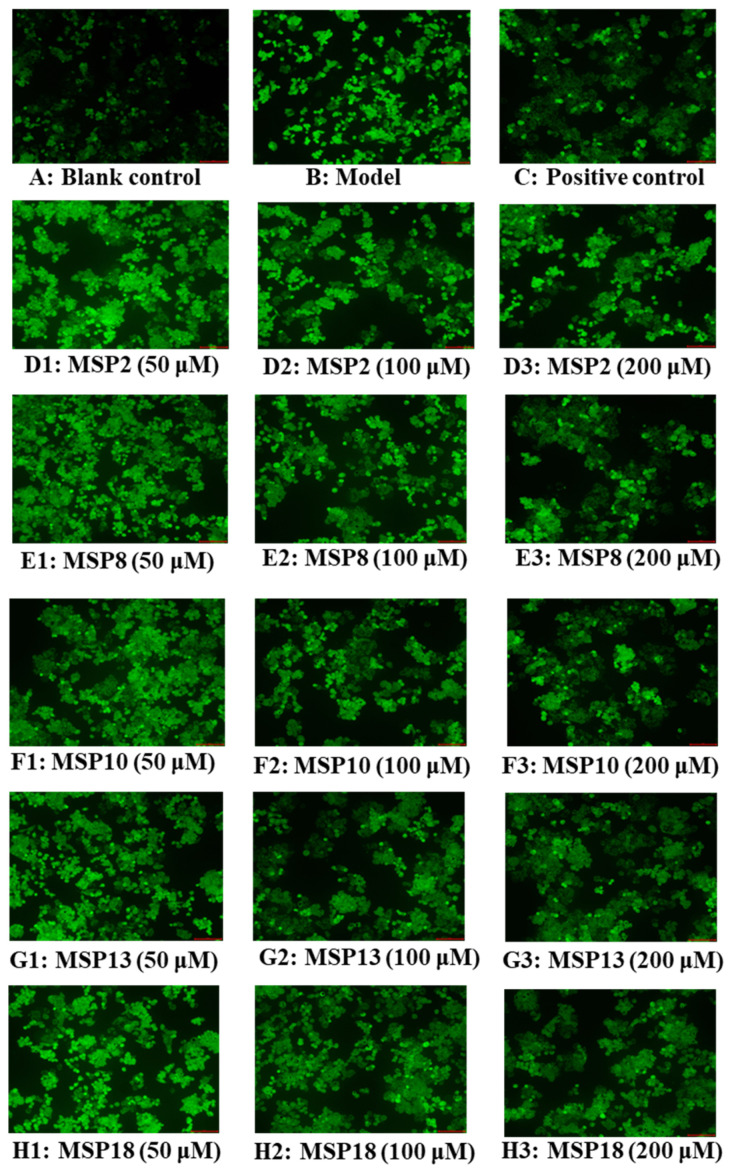
Determination of ROS levels in HepG2 cells by DCFH-DA staining (20×): (**A**) blank control; (**B**) FFA-induced cell model; (**C**) positive control (NAC); (**D1**–**D3**) MSP2 with the concentrations of 50, 100 and 200 μM, respectively; (**E1**–**E3**) MSP8 with the concentrations of 50, 100 and 200 μM, respectively; (**F1**–**F3**) MSP10 with the concentrations of 50, 100 and 200 μM, respectively; (**G1**–**G3**) MSP13 with the concentrations of 50, 100 and 200 μM, respectively; (**H1**–**H3**) MSP18 with the concentrations of 50, 100 and 200 μM, respectively.

**Figure 8:**
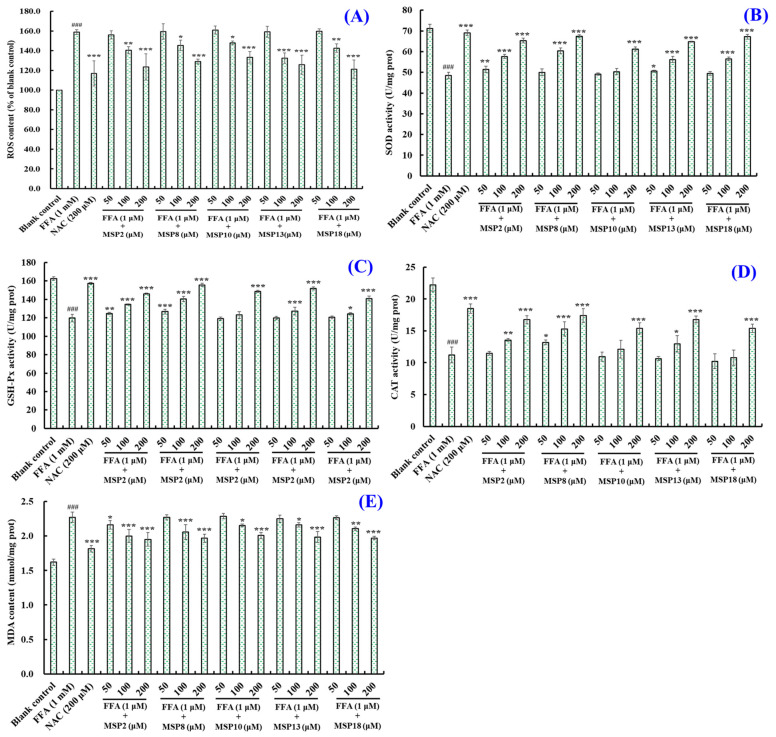
Effects of MSP2, MSP8, MSP10, MSP13 and MSP18 on ROS (**A**), SOD (**B**), GSH-PX (**C**), CAT (**D**) and MDA (**E**) levels of FFA-induced NAFLD model of HepG2 cells at 50, 100 and 200 µM. NAC at 200 µM was served as the positive control. All data are presented as the mean ± SD of triplicate results. ^###^ *p* < 0.001 vs. blank control group; *** *p* < 0.001, ** *p* < 0.01 and * *p* < 0.05 vs. FFA-induced NAFLD model of HepG2 cells.

The influences of MSP2, MSP8, MSP10, MSP13 and MSP18 on the ROS levels in the FFA-induced NAFLD model of HepG2 cells are displayed in Figure 7 and Figure 8A. Compared with the blank group (Figure 8A), the fluorescence intensity and area increased significantly due to the lipid accumulation in the model group (Figure 7B), illustrating a significant increase of intracellular ROS levels. Conversely, the increased fluorescence area and intensity induced by FFA was reduced by MSP2, MSP8, MSP10, MSP13 and MSP18, indicating a notable reduction in intracellular ROS production. Figure 8A precisely quantifies the effects of MSP2, MSP8, MSP10, MSP13 and MSP18 on the ROS levels in the FFA-induced NAFLD model of HepG2 cells. The results indicate that ROS levels were significantly reduced by MSP2, MSP8, MSP10, MSP13 and MSP18 pretreatment at medium (100 µM) and high (200 µM) concentrations in comparison to the model group (*p* < 0.001). At high concentrations, the ROS levels of the MSP2, MSP8, MSP10, MSP13 and MSP18 groups at 200 μM decreased from 158.97 ± 2.38 to 123.590 ± 13.28%, 128.950 ± 2.48%, 133.430 ± 5.89%, 125.830 ± 9.8% and 121.120 ± 9.52% of the control group, respectively. 

Figure 8B,D show that the activity of intracellular antioxidases (SOD, GSH-Px and CAT) in the FFA-induced HepG2 cells incubated with MSP2, MSP8, MSP10, MSP13 and MSP18 strengthened gradually with peptide concentrations ranging from 50 μM to 200 μM. At 200 μM, the SOD activity in the MSP2, MSP8, MSP10, MSP13 and MSP18 groups increased significantly from 48.48 ± 1.51 U/mg prot to 65.30 ± 1.16, 67.39 ± 0.69, 61.28 ± 1.02, 64.76 ± 0.23 and 67.19 ± 1.19 U/mg prot, respectively (*p* < 0.001); the GSH-Px activity in the MSP2, MSP8, MSP10, MSP13 and MSP18 groups increased significantly from 119.77 ± 4.17 U/mg prot to 146.10 ± 0.74, 155.69 ± 1.82, 148.75 ± 1.14, 151.88 ± 1.63 and 140.99 ± 2.60 U/mg prot, respectively (*p* < 0.001); and the CAT activity in the MSP2, MSP8, MSP10, MSP13 and MSP18 groups increased significantly from 11.20 ± 1.24 U/mg prot to 16.74 ± 0.66, 17.41 ± 1.09, 15.39 ± 0.86, 16.79 ± 0.54 and 15.39 ± 0.67 U/mg prot, respectively (*p* < 0.001). 

Figure 8E shows that the production of MDA of the FFA-induced HepG2 cells in the MSP2, MSP8, MSP10, MSP13 and MSP18 groups gradually decreased with concentrations ranging from 50 μM to 200 μM. At 200 μM, the production of the MDA of the FFA-induced HepG2 cells in the MSP2, MSP8, MSP10, MSP13 and MSP18 groups decreased to 1.95 ± 0.10, 1.97 ± 0.06, 2.01 ± 0.04, 1.98 ± 0.08 and 1.97 ± 0.03 nmol/mg prot, respectively, which were significantly less than the MDA production in the model group (2.27 ± 0.07 nmol/mg prot) (*p* < 0.001). Under the treatment of MSP2, MSP8, MSP10, MSP13 and MSP18, the ROS and MDA content in the NAFLD model cells decreased significantly, and intracellular antioxidant enzyme activity increased significantly. This result indicates that MSP2, MSP8, MSP10, MSP13 and MSP18 can effectively inhibit the oxidative stress response of cells and achieve the effect of interfering with NAFLD.

### 2.5. Effects of MSP2, MSP8, MSP10, MSP13 and MSP18 on the Protein Expression Related to Intracellular Lipid Metabolism and Antioxidant System

#### 2.5.1. Effects of MSP2, MSP8, MSP10, MSP13 and MSP18 on Proteins Expression Related to Lipid Metabolism

**Figure 9:**
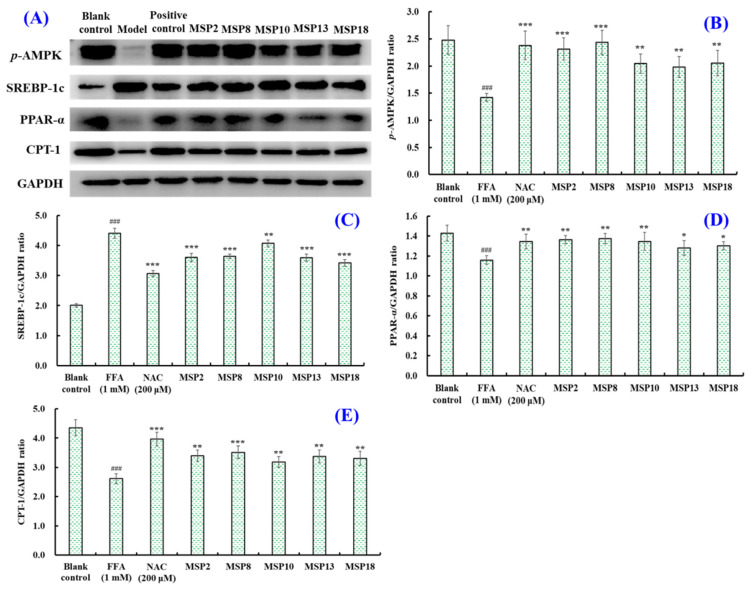
The effects of MSP2, MSP8, MSP10, MSP13 and MSP18 at a concentration of 200 μM on the expression of lipid metabolism-related proteins in FFA-induced NAFLD model in HepG2 cells. NAC at 200 µM was served as the positive control. All data are presented as the mean ± SD of triplicate results. (**A**) Expression levels of lipid metabolism related proteins; (**B**) p-AMPK/GAPDH ratio; (**C**) SREBP-1c/GAPDH ratio; (**D**) PPAR-α/GAPDH ratio; (**E**) CPT-1/GAPDH ratio. ^###^ *p* < 0.001 vs. blank control group; *** *p* < 0.001, ** *p* < 0.01 and * *p* < 0.05 vs. FFA-induced NAFLD model of HepG2 cells.

AMP-activated protein kinase (AMPK) is a key regulator of biological energy metabolism and plays an important role in mediating liver adipogenesis. When AMPK is phosphorylated, the expression level of sterol regulatory element binding proteins (SREBPs) can be directly inhibited, thus reducing the regeneration of endogenous fat and reducing the lipid accumulation in liver. In addition, the peroxisome proliferator-activated receptor α (PPAR-α) and carnitine palmitoyltransferase 1 (CPT-1) can be regulated to promote the β-oxidation of fatty acids, thus reducing the level of intracellular lipids [11,46,47]. Resultantly, the expression of lipid metabolism-linked proteins including p-AMPK, SREBP-1c, PPAR-α and CPT-1 was investigated in the FFA-induced NAFLD model of HepG2 cells to further explore the molecular mechanisms of MSP2, MSP8, MSP10, MSP13 and MSP18 inhibiting lipid accumulation (Figure 9). In the FFA-induced NAFLD model of HepG2 cells, the expression levels of the p-AMPK, PPAR-α and CPT-1 proteins significantly decreased, but the expression level of the SREBP-1c proteins significantly increased in comparison to the blank group (*p* < 0.05). What is exciting is that the expression of the lipid metabolism-linked proteins in 200 µM of MSP2, MSP8, MSP10, MSP13 and MSP18-pretreated HepG2 cells was significantly reversed, which elucidates the underlying mechanism of the hypolipidemic effects of MSP2, MSP8, MSP10, MSP13 and MSP18 (Figure 9). Compared with the model group, MSP2 and MSP8 can dramatically reduce the relative expression of the p-AMPK protein (*p* < 0.001); MSP2, MSP8, MSP13 and MSP18 can significantly reduce the relative expression of the SREBP-1c protein (*p* < 0.001); and MSP2, MSP8 and MSP10 can significantly enhance the relative expression of the PPAR-α protein (*p* < 0.01). In addition, all the peptides increased the expression of the CPT-1 protein, but MSP8 showed the strongest ability to increase the expression of CPT-1 proteins among the five peptides (*p* < 0.001) (Figure 9).

#### 2.5.2. Effects of MSP2, MSP8, MSP10, MSP13 and MSP18 on the Protein Expression 

##### Related to Intracellular Antioxidant System

**Figure 10:**
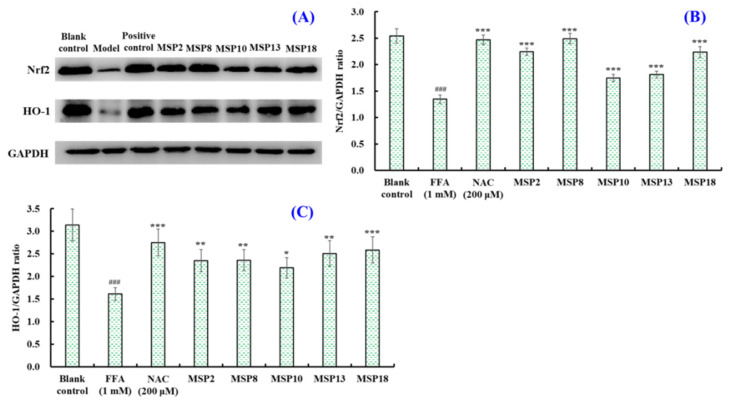
The effects of MSP2, MSP8, MSP10, MSP13 and MSP18 at 200 μM on the expression of antioxidant-system-related proteins in FFA-induced NAFLD model in HepG2 cells. NAC at 200 µM was served as the positive control. All data are presented as the mean ± SD of triplicate results. (**A**) Expression levels of antioxidant-system-related proteins; (**B**) Nrf2/GAPDH ratio; (**C**) HO-1/GAPDH ratio. ^###^ *p* < 0.05 vs. blank control group; *** *p* < 0.01, ** *p* < 0.01 and * *p* < 0.05 vs. FFA-induced NAFLD model of HepG2 cells.

Nuclear factor erythroid 2-related factor 2 (Nrf2) is one of the important antioxidant stress-signaling pathways in vivo, and heme oxygenase-1 (HO-1) is the downstream target protein regulated by Nrf2, which plays an important role in the occurrence and development of liver diseases. In addition, the expression of downstream antioxidative enzymes (CAT, SOD, GSH-PX, etc.) is up-regulated according to the accumulation of Nrf2 in the nucleus. Figure 10 indicates that the expression of the Nrf2 protein was significantly lowered in the FFA-induced NAFLD model of HepG2 cells (*p* < 0.001), which further led to a sharp drop in the heme oxygenase 1 (HO-1) protein (*p* < 0.001). However, the decrease in the expression of Nrf2 and HO-1 proteins could be significantly reversed by adding 200 µM of MSP2, MSP8, MSP10, MSP13 and MSP18 into the FFA-induced NAFLD model of HepG2 cells. In addition, MSP8 and MSP18 presented the strongest ability to promote Nrf2 and HO-1 protein expression, respectively.

## 3. Discussion

NAFLD is a complex disease whose pathogenesis has not been fully elucidated. Presently, the “two-hit” model is one of the dominant theories to discuss NFALD [48]. In brief, lipid deposition in a liver causes hyperglycosemia and hyperlipemia, and the hepatic cells initially develop steatosis, which is called the “first hit”. The “second hit” is thought to be the subsequent oxidative stress, inflammation and fibrosis it causes. Modern unhealthy lifestyles, especially high-fat diets and a lack of exercise, lead to a large accumulation of lipids in the human body. The excess FFAs overloaded in the body are transferred from the bloodstream to the liver, which result in continuous lipid accumulation and a disorder of lipid synthesis and metabolism in the liver, and thereby facilitate the development of non-alcoholic fatty livers [4,8]. Moreover, the accumulation of excess FFA in the liver induces consecutive oxidative stress reactions, leading to a production of excess ROS, immune cell polarization infiltration and a massive release of inflammatory cytokines [8,49]. As a consequence, heightened oxidative stress and immune/inflammatory responses further cause liver cell damage and liver cirrhosis and even develop into NAFLD. Thus, reducing lipid deposition and oxidative stress levels in the liver is essential for ameliorating NAFLD [8,50]. Therefore, according to the “two-hit” model, we designed this experiment to discuss the mechanisms of MSP2, MSP8, MSP10, MSP13 and MSP18 in ameliorating NAFLD from the aspect of lowering lipid accumulation and oxidative stress levels.

### 3.1. Mechanisms of MSP2, MSP8, MSP10, MSP13 and MSP18 on Ameliorating Lipid Metabolism

Hepatocyte steatosis is an important cause of fatty liver. The main cause of hepatic steatosis is that the oxidation rate of lipid is far less than the synthesis rate, and a large amount of lipid is accumulated in the liver, resulting in a disorder of lipid metabolism, steatosis and ultimately the generation of a fatty liver [8,11]. Therefore, we established the FFA-induced NAFLD model of HepG2 cells using 1.0 mM FFA for exploring the ameliorating activity and mechanisms of MSP1–MSP18 on NAFLD. 3-(4,5-dimethylthiazolyl-2)-2, 5-diphenyltetrazolium bromide (MTT), O staining and the TG and TC content-determination assays proved that the FFA-induced NAFLD model of HepG2 cells is suitable for studying the activity and mechanisms of MSP1–MSP18 (Figure 2 and Figure 3A). The effects of MSP1–MSP18 on lipid accumulation in the NAFLD cell model were investigated, and MSP2, MSP8, MSP10, MSP13 and MSP18 presented significant lipid lowering effects, demonstrating the significantly ameliorating functions of MSP2, MSP8, MSP10, MSP13 and MSP18 on FFA-induced HepG2 cells (Figure 5, Figure 6 and Figure 7).

**Figure 11:**
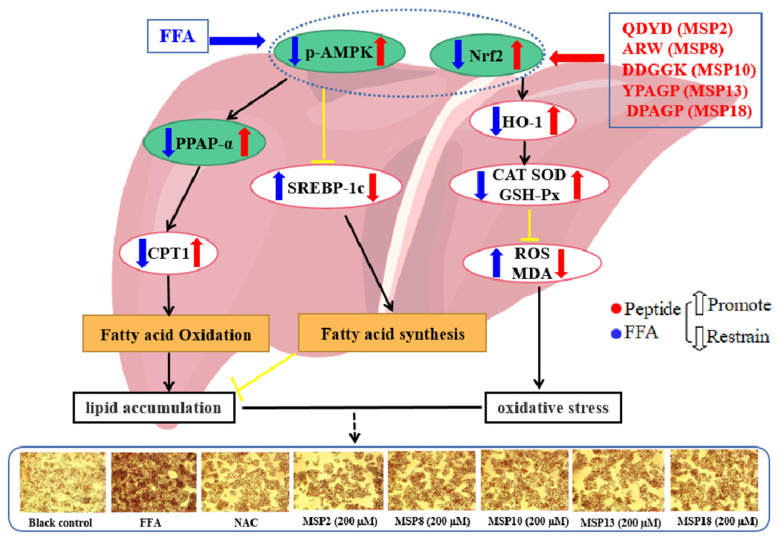
Mechanisms of MSP2, MSP8, MSP10, MSP13 and MSP18 on ameliorating NAFLD by suppressing lipid accumulation and oxidative stress via the AMPK/Nrf2 pathway.

AMPK is a cellular energy sensor that plays a vital role in maintaining human energy homeostasis [51]. AMPK is a serine/threonine protein kinase that is activated by low energy states. Consequently, the activated AMPK pathway can catalyze the production of large amounts of ATP to replenish consumption during anabolic processes and restore the energy balance of cells [52]. In addition, the biological activity of AMPK is regulated by the phosphorylation or dephosphorylation of upstream kinases and phosphatases [53]. In livers, AMPK phosphorylation (p-AMPK) can regulate lipid-related transcription factors, such as SREBP-1c, PPAR-α and CPT-1, to reduce lipid accumulation [54]. Thus, the AMPK pathway is involved in positive lipid regulation in livers and is identified as a key therapeutic target for NAFLD. As an important regulator of lipid homeostasis, SREBPs play a key role in de novo fat synthesis and are tightly relevant to the occurrence of NAFLD [55]. SREBP-1c is one of the subtypes of SREBPs and is mainly expressed in the liver and adipose tissue. Overexpression of SREBP-1c can promote the expression of downstream target genes, thus up-regulating TG synthesis and promoting the occurrence of steatosis [56,57]. PPAR-α is mainly expressed in tissues with high fatty acid catabolism, including the liver, heart and kidney. PPAR-α is involved in fatty acid oxidation in the liver through significantly up-regulated gene expression and can affect the development of NAFLD and NASH [58,59]. CPT-1 is a downstream protein of PPAR-α and a key rate-limiting enzyme of fatty acid oxidation in the liver. CPT-1 can promote β-oxidation to reduce intracellular FFA and inhibit the secretion of cellular pro-inflammatory factors [60,61]. Therefore, regulating lipid metabolism through the AMPK pathway is considered a feasible therapeutic strategy to prevent the occurrence and development of NAFLD. In order to further elucidate lipid-lowering mechanisms, we determined the effects of MSP2, MSP8, MSP10, MSP13 and MSP18 on the expression of the *p*-AMPK, SREBP-1c, PPAR-α and CPT-1 proteins. The Western blot results proved that MSP2, MSP8, MSP10, MSP13 and MSP18, especially MSP2 and MSP8, can significantly promote AMPK phosphorylation, inhibiting the expression level of the lipid synthesis factor of SREBP-1c to reduce the production of lipids. Moreover, MSP2, MSP8, MSP10, MSP13 and MSP18, especially MSP2, MSP8 and MSP10, can significantly promote the expression of the PPAR-α and CPT-1 proteins to accelerate the β-oxidation of fatty acids (Figure 11). These results demonstrate that the lipid lowering mechanisms of MSP2, MSP8, MSP10, MSP13 and MSP18 are involved in the regulation of lipid metabolism via regulating the AMPK pathway and its downstream protein factors on lipid synthesis (SREBP-1c) and degradation (PPAR-α and CPT-1) (Figure 11).

### 3.2. Mechanisms of MSP2, MSP8, MSP10, MSP13 and MSP18 on Regulating Intracellular Antioxidant System

Oxidative stress is an important mechanism of the NAFLD transmission of liver injuries [48]. In NAFLD patients, oxidized FFAs enter the liver and destroy the mitochondrial electron transport chain and intracellular antioxidant system, which further leads to the decrease of the activities of intracellular antioxidant enzymes, promotes the mass production of ROS, increases the production of lipid peroxide MDA and causes oxidative stress [6,49]. The produced MDA further induces the cross-linking polymerization of macromolecules and has a toxic effect on cells. In addition, oxidative stress can also activate inflammatory pathways and cause mitochondrial dysfunction. These destructive effects lead to the deterioration of NAFLD into NASH [49,62]. Therefore, we determined the activity of intracellular antioxidant protease (CAT, SOD and GSH-PX) and the content of lipid peroxides (MDA). The findings show that MSP2, MSP8, MSP10, MSP13 and MSP18 can significantly reduce ROS content, significantly increase SOD, GSH-PX and CAT levels and reduce the MDA content in FFA-induced HepG2 cells (Figure 7 and Figure 8). It was proven that MSP2, MSP8, MSP10, MSP13 and MSP18 had strong cytoprotection for HepG2 cells against FFA-induced oxidative damage.

The Nrf2 pathway is the most important endogenous antioxidant stress pathway, which can regulate the expression of antioxidants and detoxification enzymes and is the main regulatory factor of antioxidants [63,64]. Nrf2 has also been proven to be a potential target for curing NAFLD [65,66]. The literature shows that Nrf2 agonists can regulate the expression of the downstream product HO-1 protein by activating the Nrf2 pathway and then up-regulate the activities of antioxidant proteases to remove excess ROS and reduce oxidative stress damage and further affect the liver lipid metabolism and inflammatory response to avoid liver damage and prevent the occurrence of NASH [49]. Therefore, HO-1 has also been considered as an important target for treating metabolic diseases [67]. In order to clarify the antioxidant mechanisms of MSP2, MSP8, MSP10, MSP13 and MSP18, we determined the expression levels of the oxidative stress-related proteins Nrf2 and HO-1. The present findings show that MSP2, MSP8, MSP10, MSP13 and MSP18 can up-regulate the expression of Nrf2 and HO-1 proteins to activate the Nrf2 pathway, which further regulates the expression of downstream antioxidant protease (SOD, GSH-PX and CAT) to remove excess ROS, reduce the content of MDA and eventually alleviate cellular oxidative stress response (Figure 11).

## 4. Materials and Methods

### 4.1. Materials and Reagents

The Dulbecco’s Modified Eagle Medium (DMEM), protein Marker (11–180 KDa), penicillin-streptomycin solution, L-Glutamine, glutathione (GSH), phosphate buffered saline (PBS), RPMI-1640, fetal bovine serum (FCS), MTT and trypsin-EDTA assay kit were purchased from Beijing Solarbio Technology Co., Ltd. (Beijing, China). Antibodies of *p*-AMPK, SREBP-1c, PPAR-α and CPT-1 were purchased from Affinity Biopharmaceutical Co., Ltd. (Shanghai, China). Antibodies of HO-1, Nrf2 and GAPDH were purchased from Proteintech Group, Inc. (Wuhan, China). Assay kits for measuring the activity of SOD, CAT and GSH-Px and contents of TG, TC, BCA, ROS and MDA were purchased from Nanjing Jiancheng Bioengineering Institute (Nanjing, China). Oil red O solution, NAC, palmitic acid (PA), oleic acid (OA) and β-actin were purchased from Sigma-Aldrich (Shanghai, China) Trading Co., Ltd. (Shanghai, China). Eighteen antioxidant peptides (MSP1–MSP18) (>98%) were synthesized by Shanghai Apeptide Co., Ltd. (Shanghai, China).

### 4.2. HepG2 Cell Culture and Establishment of NAFLD Cell Model

The FFA-induced NAFLD cell model was established according to previous methods [68,69]. HepG2 cells were cultured in DMEM containing 10% FBS and 1% penicillin-streptomycin at 37 °C and 50 mL/L CO_2_ in a 95% humidified sterile environment. 

HepG2 cells in the logarithmic growth phase were seeded into 96-well plates and cultivated continuously for 24 h. The cell medium was replaced after the cell density increased to 80% in different concentrations (0.25, 0.5, 1.0, 1.5 and 2.0 mM) of FFA solution (PA and OA mixture with a molar ratio of 1:2 containing 1% BSA), amidst a 200 mL culture medium for each well. After culturing for 24 h, the cultured HepG2 cells were observed and photographed using a microscope.

### 4.3. Cells Viability Determination

Cells’ viability was determined using an MTT assay [21]. In brief, 100 μL of the peptide sample at designed concentrations (50, 100, or 200 μM) and 100 μL of growth media were joined in the HepG2 cells. The cultures in the blank control group were replaced by PBS without peptides. Cell viability (% of blank control) was determined after HepG2 cells were incubated for 24 h.

### 4.4. Oil Red O Staining Assay

The assay was carried out according to the manufacturer’s instructions of the Oil red O staining kit [11]. HepG2 cells were fixed on 96-well plates with 4% formaldehyde for 0.5 h and cleaned with PBS twice. After that, HepG2 cells were rinsed with 60% isopropanol for 10 min, and the isopropanol was cleared up. Then, 3% Oil red O solution was added into 96-well plates and incubated with the HepG2 cells for 1 h, and the cells were rinsed with PBS three times to clear away the free dye. Finally, stained cells were added to DMSO, and the absorbance at 358 nm was measured. The stained cells were photographed using an inverted microscope, Olympus IX71 (Olympus Co., Ltd., Shinjuku, Japan).

### 4.5. Protein Extraction of HepG2 Cells

HepG2 cells were washed by PBS two times and dealt with a lysis buffer (1% Triton X-100, 1% deoxycholate and 0.1% SDS) on ice for 20 min. Subsequently, the mixed solution was centrifuged at 12,000× *g* at 4 °C for 20 min. The BCA protein assay kit was used to measure protein concentration according to the manufacturer’s instructions.

### 4.6. Intracellular TC, TG, MDA, and Antioxidant Enzymes Level Analysis

HepG2 cells were seeded into 96-well plates and treated with the processes described in Section 4.3, Section 4.4 and Section 4.5. Intracellular TC and TG contents were measured using their assay kits according to the manufacturer’s instructions [11].

The levels of SOD, GSH-Px, CAT and MDA were determined following the instructions of the manufacturers [70,71]. 

### 4.7. Intracellular ROS Level Analysis

The ROS level was determined by the DCFH-DA staining method [72,73]. Briefly, the cells were treated according to the above method (4.3 to 4.5) and washed with PBS three times. After that, the HepG2 cells were incubated with 10 μM DCFH-DA for 30 min at 37 °C. After washing with PBS two times, the morphology of the HepG2 cells was observed and photographed using an inverted microscope (Nikon Corporation, Kyoto, Japan), and the fluorescence of the cells was monitored at 485 nm excitation (535 nm emission).

### 4.8. Western Blot Assay 

The procedure of Western blotting was performed according to reported methods [25,74]. The BCA protein assay kit was used to measure the protein concentration according to the manufacturer’s instructions. Extracted proteins were loaded onto 12% SDS-PAGE and, in the next step, were transferred onto a PVDF membrane. After incubating with primary and secondary antibodies, protein bands were visualized with enhanced chemiluminescence (ECL), photographed and analyzed quantitatively with the FluorChem FC3 software (Bio-Techne, Minneapolis, MN, USA). 

### 4.9. Statistical Analysis

All the data are expressed as the mean ± SD (*n* = 3). The experimental data were analyzed by an ANOVA test using SPSS 19.0. Significant differences were determined by Duncan’s multiple range test (*p* < 0.05, 0.01, and 0.001).

## 5. Conclusions

In summary, we systematically studied the alleviating functions and mechanisms of MSP2, MSP8, MSP10, MSP13 and MSP18 on an FFA-induced NAFLD model of HepG2 cells through inhibiting lipid accumulation and oxidative stress. The lipid-lowering mechanisms of MSP2, MSP8, MSP10, MSP13 and MSP18 firstly demonstrated that they can ameliorate lipid metabolic disorders in vitro through regulating the AMPK pathway and its downstream protein factors on lipid synthesis (SREBP-1c) and degradation (PPAR-α and CPT-1). In addition, the mechanisms of MSP2, MSP8, MSP10, MSP13 and MSP18 on inhibiting cellular oxidative stress response revealed that they can remove excess ROS and reduce the content of MDA by activating the Nrf2 pathway to up-regulate the expression levels of the HO-1 protein and downstream antioxidant protease (SOD, GSH-PX and CAT). Therefore, the present findings provide a good perspective on antioxidant peptides with monkfish swim bladders acting as antioxidant ingredients applied in health-promoting products on NAFLD. 

## Data Availability

Data are contained within the article.

## Abbreviations

AMPK, AMP-activated protein kinase; CAT, catalase; CPT-1, Carnitine palmitoyl transterase-1; DMEM, Dulbecco’s Modified Eagle Medium; FFA, free fatty acid; GSH, L-Glutathione; GSH-Px, glutathione peroxidase; HO-1, heme oxygenase 1; MDA, malondialdehyde; NAC, N-Acetyl-L-cysteine; NAFLD, non-alcoholic fatty liver disease; Nrf2, nuclear factor erythroid 2-related factor 2; PPAR, peroxisome proliferator-activated receptor; ROS, reactive oxygen species; SOD, superoxide dismutase; SREBP, sterol regulatory element binding proteins; TC, total cholesterol; TG, triglyceride.
